# Reorientational Dynamics in Y(BH_4_)_3_·*x*NH_3_ (*x* = 0, 3, and 7): The Impact of NH_3_ on BH_4_^–^ Dynamics

**DOI:** 10.1021/acs.jpcc.4c00265

**Published:** 2024-03-09

**Authors:** J. B. Grinderslev, U. Häussermann, T. R. Jensen, A. Faraone, M. Nagao, M. Karlsson, T. J. Udovic, M. S. Andersson

**Affiliations:** 1 Interdisciplinary Nanoscience Center (iNANO) and Department of Chemistry, Aarhus University, Aarhus DK-8000, Denmark; 2 Department of Materials and Environmental Chemistry, Stockholm University, SE-10691 Stockholm, Sweden; 3 NIST Center for Neutron Research, National Institute of Standards and Technology, Gaithersburg, Maryland 20899-6102, United States; 4 Department of Materials Science and Engineering, University of Maryland, College Park, Maryland 20742-2115, United States; 5 Department of Physics and Astronomy, University of Delaware, Newark, Delaware 19716, United States; 6 Department of Chemistry and Chemical Engineering, Chalmers University of Technology, Göteborg SE-412 96, Sweden; 7 Ångström Laboratory, Department of Chemistry, Uppsala University, Box 538, SE-751 21 Uppsala, Sweden

## Abstract

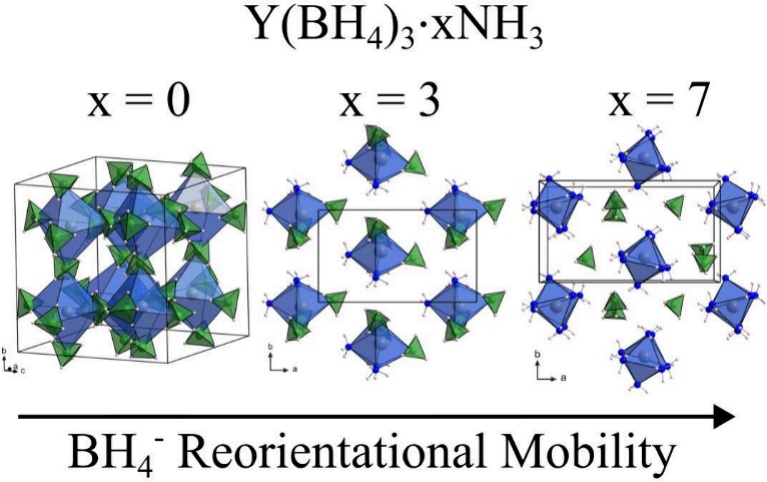

The reorientational dynamics of Y(BH_4_)_3_·*x*NH_3_ (*x* = 0, 3, and 7) was studied using quasielastic neutron scattering (QENS) and neutron spin echo (NSE). The results showed that changing the number of NH_3_ ligands drastically alters the reorientational mobility of the BH_4_^–^ anion. From the QENS experiments, it was determined that the BH_4_^–^ anion performs 2-fold reorientations around the C_2_ axis in Y(BH_4_)_3_, 3-fold reorientations around the C_3_ axis in Y(BH_4_)_3_·3NH_3_, and either 2-fold reorientations around the C_2_ axis or 3-fold reorientations around the C_3_ axis in Y(BH_4_)_3_·7NH_3_. The relaxation time of the BH_4_^–^ anion at 300 K decreases from 2 × 10^–7^ s for *x* = 0 to 1 × 10^–12^ s for *x* = 3 and to 7 × 10^–13^ s for *x* = 7. In addition to the reorientational dynamics of the BH_4_^–^ anion, it was shown that the NH_3_ ligands exhibit 3-fold reorientations around the C_3_ axis in Y(BH_4_)_3_·3NH_3_ and Y(BH_4_)_3_·7NH_3_ as well as 3-fold quantum mechanical rotational tunneling around the same axis at 5 K. The new insights constitute a significant step toward understanding the relationship between the addition of ligands and the enhanced ionic conductivity observed in systems such as LiBH_4_·*x*NH_3_ and Mg(BH_4_)_2_·*x*CH_3_NH_2_.

## I Introduction

Metal borohydrides are a fascinating and continuously expanding class of materials, and their extremely rich chemistry, including a wide range of compositions and structural flexibility, has resulted in a plethora of new materials in the past decade.^1−4^ These new materials exhibit a wide variety of interesting properties such as luminescence, magnetism, semiconductivity, and superionic conductivity.^5−10^ The interest in metal borohydrides as superionic conductors was initiated by the discovery of fast Li^+^ conductivity in the high-temperature polymorph of LiBH_4_ in 2007.^11^ Since then, there have been numerous reports on strategies to improve the ionic conductivity at lower temperatures, often by cation or anion substitution, nanostructuring, or nanocomposite formation.^12,13^ More recently, metal borohydride derivatives with neutral ligands, such as LiBH_4_ coordinated with different neutral molecules (H_2_O, NH_3_, CH_3_NH_2_, and NH_3_BH_3_), have received increased attention and have demonstrated the highest reported ionic conductivities among LiBH_4_-based conductors.^14−19^ Likewise, this strategy has also proven fruitful for multivalent (e.g., Mg^2+^) ionic conductors, and the highest solid-state Mg^2+^ conductivities are reported for Mg(BH_4_)_2_ derivatives with neutral ligands such as NH_3_, CH_3_NH_2_, NH_3_BH_3_, (CH_3_)_2_CHNH_2_, NH_2_CH_2_CH_2_NH_2_, and O(CH_2_)_4_.^20−27^ The underlying mechanism behind this new type of ionic conductors is still not completely understood, but the flexible structural framework and versatile coordination of BH_4_^–^ appear to be crucial, and an exchange of the neutral molecule between framework and interstitial cations may promote the cationic conductivity.^4,17,21^ Dynamic studies on other related materials have shown that BH_4_^–^ may actively promote the ionic conductivity through rapid reorientations as reported for the LiLa(BH_4_)_3_X (X = Cl, Br, I) and LiBH_4_–LiI systems.^28−31^ Likewise, rapid BH_4_^–^ dynamics were also identified in the fast Li^+^ ion conductor LiBH_4_·NH_3_.^32,33^ Thus, the dynamics of BH_4_^–^ play an important role for physical properties such as high cation conductivity.^33^ A related class of materials with larger boron–hydrogen cluster anions are also receiving significant attention, where the rapid reorientation of the polyhedral anions in M_2_B_*x*_H_*x*_ and MCB_*x*–1_H_*x*_ (M = Li, Na, and K; *x* = 10 and 12) plays an important part in the superionic conductivities observed in these systems.^34^

In general, introducing a neutral ligand opens the structures, allowing for new interstitial sites and conduction pathways.^4^ A good example of this is the ammine yttrium borohydrides, which have a high compositional variety, Y(BH_4_)_3_·*x*NH_3_ (*x* = 1, 2α, 2β, 3, 5, 6, 7), where the three-dimensional structure of Y(BH_4_)_3_ breaks down to two-dimensional layers (*x* = 1), one-dimensional chains (*x* = 2), molecular units (*x* = 3), and separate ionic complexes (*x* = 5, 6, 7); see Figure S1 in the Supporting Information.^35,36^ This has prompted the present investigation, where we study the effect on the reorientational properties of BH_4_^–^ and NH_3_ and how it is affected by changes in the structural framework and resulting local coordination as the ammonia content is increased from *x* = 0 to 3 and 7 in Y(BH_4_)_3_·*x*NH_3_.

## II Methods

### II.A Synthesis

Y(^11^BH_4_)_3_ was prepared using a slightly modified approach compared to previously published procedures.^8,37^ Y-metal (99.9%, Sigma-Aldrich^38^) was hydrogenated by heating from room-temperature to 400 °C, with a heating rate of 5 K/min and an initial H_2_ pressure of 140 bar. The samples were subsequently cooled to room temperature, after which the pressure was released. The resulting YH_3_ was ball-milled using a Fritsch Pulverisette no. 6 in an 80 mL tungsten-carbide vial together with tungsten-carbide-coated steel balls (*d* = 10 mm) in a ball-to-powder mass ratio of 10:1, with a ball-milling program of 10 min at 350 rpm, followed by a 2 min break. This sequence was repeated 10 times. The as-milled YH_3_ was added to a round-bottomed flask with a valve outlet. Boron-11-enriched dimethyl sulfide borane (S(CH_3_)_2_·^11^BH_3_, 10 M, Katchem) was added to the powder in the molar ratio 4.5:1 (50% excess of S(CH_3_)_2_·^11^BH_3_ and diluted to a 5 M solution with toluene (anhydrous, Sigma-Aldrich). The reaction mixture was stirred at 318 K for 7 days. Subsequently, the solvent was removed by filtration, and the powder was washed twice with toluene. The dry powdered Y(^11^BH_4_)_3_·S(CH_3_)_2_ was transferred to Schlenk tubes and heated to 413 K in argon atmosphere for 2 h, followed by 2 h under dynamic vacuum (*p* ≈ 10^–4^ bar), resulting in Y(^11^BH_4_)_3_. A similar approach was used for the synthesis of Y(^11^BD_4_)_3_ using a D_2_ pressure of 50 bar and S(CH_3_)_2_·^11^BD_3_ (10 M, Katchem) instead. The resulting Y(^11^BH_4_)_3_ or Y(^11^BD_4_)_3_ was reacted with anhydrous NH_3_ or ND_3_ gas at 253 K for 2 h. The white powders were identified by powder X-ray diffraction (PXD) as Y(^11^BH_4_)_3_·7NH_3_, Y(^11^BD_4_)_3_·7NH_3_, Y(^11^BH_4_)_3_·7ND_3_, or Y(^11^BD_4_)_3_·7ND_3_. Y(^11^BH_4_)_3_·3NH_3_ and Y(^11^BH_4_)_3_·3ND_3_ were prepared by thermal treatment of the heptaamines at 333 K under vacuum for 3 h. In the remainder of this article, the isotope number for boron is omitted; however, all samples presented in this article are ^11^B-enriched.

### II.B Neutron Scattering

The neutron experiments were performed at the NIST Center for Neutron Research in the USA using the high flux backscattering spectrometer (HFBS),^39^ the time-of-flight disk chopper spectrometer (DCS),^40^ and the neutron spin echo (NSE) spectrometer. The neutron data were reduced and analyzed using DAVE.^41^ The polycrystalline powdered sample (≈0.2 g to ≈0.3 g) was evenly distributed in an aluminum foil sachet, and the thickness was kept thin (≈90% neutron transmission) to minimize multiple neutron scattering. The sachet was folded into a cylindrical shape and then slotted into a sealed cylindrical aluminum can for measurement. All of the sample preparations were done inside a glovebox with an inert atmosphere (He) to avoid sample degradation.

#### QENS

Elastic fixed window scans (EFWS) were simultaneously collected during either heating or cooling for Y(BH_4_)_3_·*x*NH_3_ (*x* = 0, 3, and 7). In an EFWS experiment, the intensity of a narrow energy slice centered at the elastic peak position is integrated for each individual temperature. On the HFBS, this is done by setting the Doppler speed to zero and integrating the number of neutron counts over a small time window (1 min) while slowly changing the temperature (≈1 K/min). As the dynamics develop on the instrumental time scale, the integrated intensity will decrease, since the quasielastic component becomes broader than the elastic peak and the total scattering intensity (sum of elastic and quasielastic) is constant.

When conducting QENS measurements, the obtained quantity is the scattering function
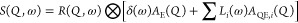
1where *E* = ℏω is the neutron energy transfer, ℏ is the Planck constant/(2π), ω is the angular frequency, δ is a delta function, *L*_*i*_’s are Lorentzian functions used to describe the quasielastic scattering, and *A*_E_ and *A*_QE,*i*_ are the areas corresponding to the respective delta and Lorentzian functions. Either one or two Lorentzians were used. The delta and Lorentzian functions are convoluted with the instrument resolution function *R*(*Q*, ω). The QENS measurements were made at several different temperatures (*T*) and/or neutron wavelengths (λ) using either HFBS or DCS. For Y(BH_4_)_3_·*x*ND_3_, QENS measurements were made at HFBS (λ = 6.271 Å) for *x* = 0 at 5 K, 410 K, 428 K, 437 K, 445 K, and 462 K and for *x* = 3 and 7 at 5 K. For Y(BH_4_)_3_·*x*ND_3_, QENS measurements were also made at DCS for *x* = 3 using a wavelength of 2.75 Å at 5 K and 232 K; for *x* = 3 using a wavelength of 4.8 Å at 5 K, 200 K, 232 K, 278 K, and 310 K; for *x* = 3 using a wavelength of 12 Å at 5 K; and for *x* = 7 using a wavelength of 4.8 Å at 5 K, 100 K, 150 K, 200 K, 250 K, and 300 K. For Y(BH_4_)_3_·*x*NH_3_, QENS measurements were made at HFBS (λ = 6.271 Å) for *x* = 3 and 7 at 5 K. For Y(BH_4_)_3_·*x*NH_3_, QENS measurements were also made at DCS for *x* = 3 using a wavelength of 2.75 Å at 5 K and 310 K; for *x* = 3 using a wavelength of 4.8 Å at 5 and 80 K; for *x* = 3 using a wavelength of 12 Å at 5 K; for *x* = 7 using a wavelength of 2.4 Å at 5 K and 150 K; and for *x* = 7 using a wavelength of 4.8 Å at 5 K and 75 K.

From the above-described QENS measurements, the elastic and quasielastic contributions were extracted by fitting the data to eq 1. Using the elastic and quasielastic contributions, the elastic incoherent structure factor (EISF) can be estimated using the following relation,
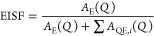
2

The experimentally determined EISF can be compared to EISF models to determine the reorientational mechanism or mechanisms of the studied compound.

#### NSE

The NSE techniques probe energy changes corresponding to dynamics in the nanosecond regime using the neutron spin precession period in a magnetic field and directly measure the intermediate scattering function *I*(*Q*, *t*), which is the time Fourier transform of *S*(*Q*, ω). NSE measurements were made at 3.5 K for Y(BH_4_)_3_·7NH_3_, Y(BH_4_)_3_·7ND_3_, and Y(BD_4_)_3_·7NH_3_ using a wavelength of 5 Å.

All structural depictions were made using the VESTA (visualization for electronic and structural analysis) or the Diamond software.^42^ For all figures, standard uncertainties are commensurate with the observed scatter in the data if not explicitly designated by vertical error bars.

## III Results and Discussion

### III.A EFWS and General Observations of the QENS Spectra

**Figure 1:**
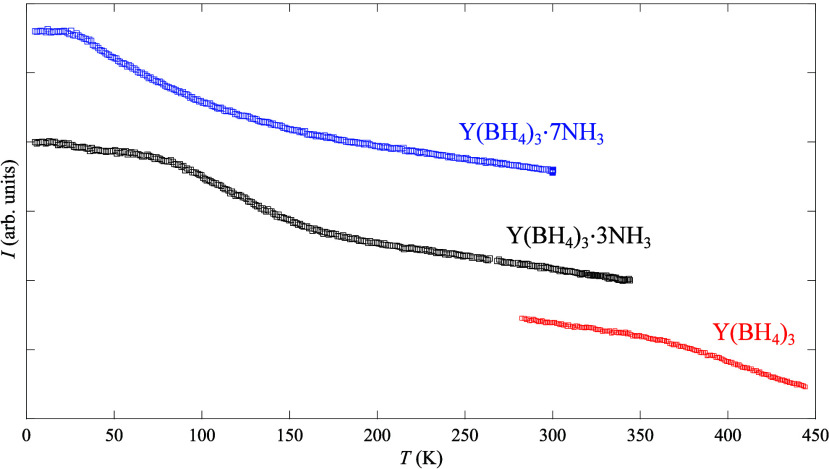
Elastic fixed window scans for Y(BH_4_)_3_, Y(BH_4_)_3_·3NH_3_, and Y(BH_4_)_3_·7NH_3_.

Figure 1 shows the EFWS for Y(BH_4_)_3_, Y(BH_4_)_3_·3NH_3_, and Y(BH_4_)_3_·7NH_3_. In the figure, it can be seen that all three EFWS curves exhibit a more or less constant slope at lower temperatures, followed by a steeper drop in the EFWS intensity at higher temperatures. For Y(BH_4_)_3_·3NH_3_ and Y(BH_4_)_3_·7NH_3_, the curve assumes a constant slope above ≈200 K, while this is not seen for Y(BH_4_)_3_ in the studied temperature range. The initial drop is due to the dynamics becoming fast enough to be observed on the instrument time scale (about 0.1 μeV ≈ 10 ns), while the change to a constant slope at higher temperatures indicates that the dynamics are now faster than the instrument time scale (about 10 μeV ≈ 0.1 ns). The observed linear decrease in the intensity with increasing temperature above and below the more significant decrease in intensity is most likely related to the Debye–Waller factor. Comparing the EFWS curves for the three samples reveals that the onset temperature of dynamics is much higher for Y(BH_4_)_3_ (about 350 K) compared to Y(BH_4_)_3_·3NH_3_ (about 80 K) and Y(BH_4_)_3_·7NH_3_ (about 25 K). However, from the EFWS data, it is not possible to say if the developing dynamics in Y(BH_4_)_3_·3NH_3_ and Y(BH_4_)_3_·7NH_3_ are related to the BH_4_^–^ and/or the NH_3_. Looking closely at the Y(BH_4_)_3_·3NH_3_, a small change in the EFWS curve can be seen between 25 and 50 K, suggesting a change in dynamics at low temperature in addition to the more significant changes in the dynamics occurring at higher temperatures.

**Figure 2:**
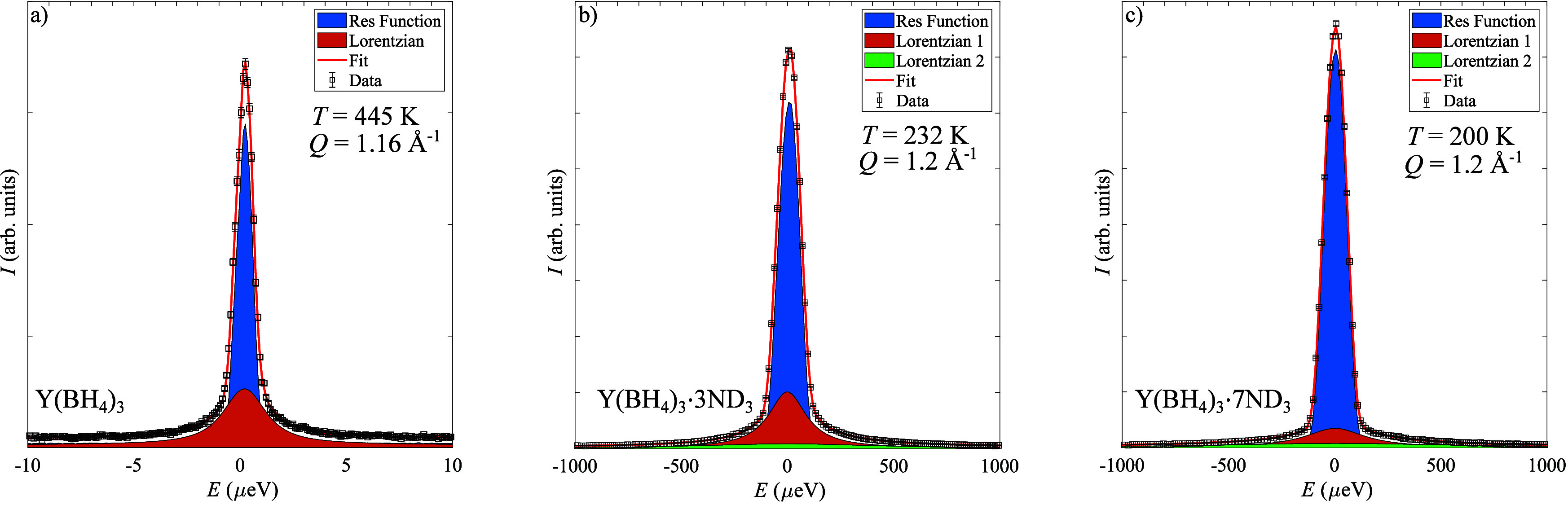
Fits of the QENS spectra showing the individual components of the fit: (a) Y(BH_4_)_3_, (b) Y(BH_4_)_3_·3ND_3_, and (c)Y(BH_4_)_3_·7ND_3_. Lorentzian 2 (green) in both parts b and c is much wider than Lorentzian 1 (red) and can thus be difficult to see. Error bars in parts a–c correspond to one standard deviation.

To determine the dynamics of the BH_4_^–^ anion, several QENS spectra were collected for Y(BH_4_)_3_, Y(BH_4_)_3_·3ND_3_, and Y(BH_4_)_3_·7ND_3_ based on temperatures suggested by the EFWS. Due to the large incoherent scattering cross section of hydrogen compared to all other elements in the samples, the QENS signal from the hydrogen in the BH_4_^–^ anion will completely dominate the spectra after deuteration of the ammonia. This means that any dynamics observed for Y(BH_4_)_3_, Y(BH_4_)_3_·3ND_3_, and Y(BH_4_)_3_·7ND_3_ are related to the BH_4_^–^ anion. Y(BH_4_)_3_, Y(BH_4_)_3_·3ND_3_, and Y(BH_4_)_3_·7ND_3_ all exhibit significant QENS broadening albeit at different temperatures, suggesting that the BH_4_^–^ anion is dynamically active in all samples. The data also reveal that the BH_4_^–^ anion dynamics become more rapid with increasing ammonia content since the width of the QENS component increases with increasing ammonia content; see Figure 2. This is in good agreement with the results from the EFWS. Fitting of the spectra to eq 1 revealed that only one Lorentzian is required to describe the scattering of BH_4_^–^ in Y(BH_4_)_3_, while two Lorentzians are needed to describe the BH_4_^–^ anion dynamics in Y(BH_4_)_3_·3ND_3_ and Y(BH_4_)_3_·7ND_3_; see Figure 2. Only one Lorentzian was needed to describe the 100 K QENS spectra for Y(BH_4_)_3_·7ND_3_. The need for two Lorentzians to describe the data suggests a distribution of the dynamics for the BH_4_^–^ anions, which could either come from a difference in the time scale of dynamical motions of the same type or from a difference in type of dynamical motion. All of the Lorentzian components have a fixed width as a function of wave vector transfer (*Q*) suggesting that the dynamics is of a local character, i.e., that the BH_4_^–^ anion performs reorientational rather than translational dynamics on the time scales probed by the spectrometers; see Figure S2 in the Supporting Information.

### III.B Reorientational Dynamics for BH_4_^–^

**Figure 3:**
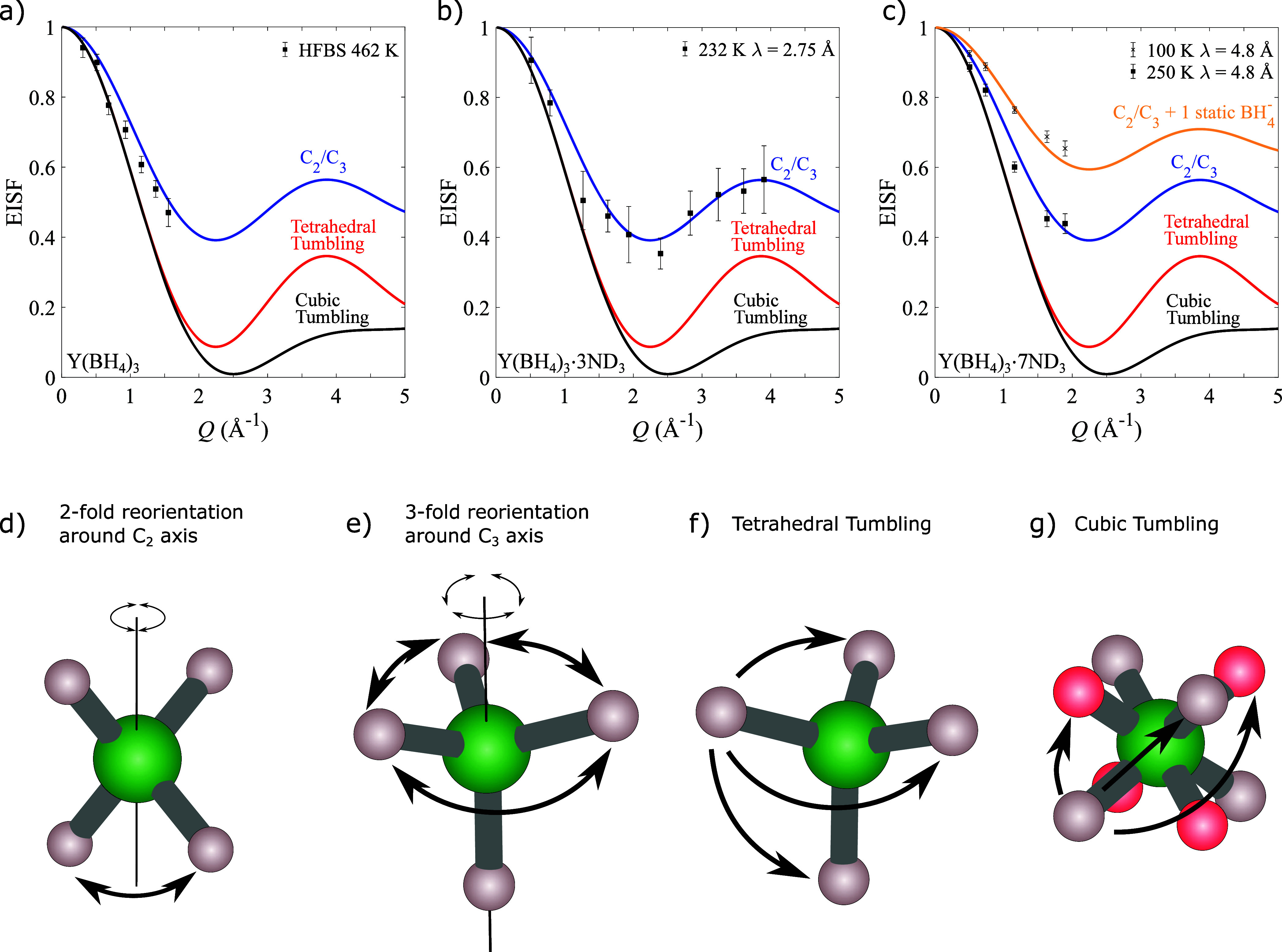
EISFs determined from QENS spectra for (a) Y(BH_4_)_3_, (b) Y(BH_4_)_3_·3ND_3_, and (c) Y(BH_4_)_3_·7ND_3_ at different temperatures compared to EISF models for each respective system. (d–f) Representations of common reorientational motions for a tetrahedral anion such as BH_4_^–^. (d) 2-fold reorientation around one of the 2-fold symmetry axes (C_2_). (e) 3-fold reorientation around one of the 3-fold symmetry axes (C_3_). (f) Tetrahedral tumbling where all of the hydrogen atoms can exchange positions with each other. This can be achieved by performing several 2-fold or 3-fold reorientations around multiple axes. (g) Cubic tumbling, where the hydrogen atoms can visit all of the corners of a cube. These corners correspond to two tetrahedra rotated by 90° around a C_2_ axis. To emphasize the positions of the two tetrahedra, the hydrogen corresponding to tetrahedron 1 have been marked as gray while the hydrogen corresponding to tetrahedron 2 have been marked as pink. Colors scheme: boron (green); hydrogen (gray/pink). Error bars in (a)–(c) correspond to two standard deviations.

**Figure 4:**
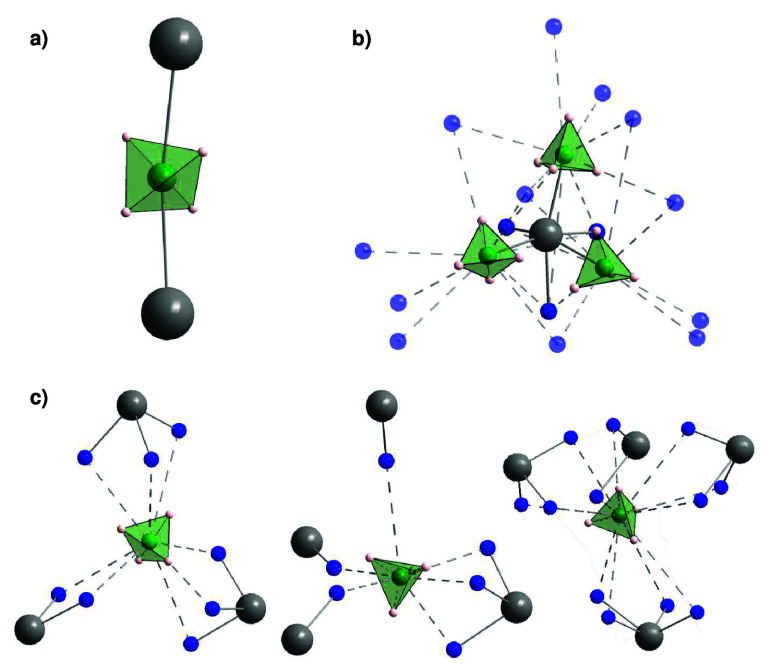
Local BH_4_^–^ environment in (a) α-Y(BH_4_)_3_, (b) Y(BH_4_)_3_·3NH_3_, and (c) Y(BH_4_)_3_·7NH_3_. Solid bonds depict the BH_4_^–^ and NH_3_ bonds toward the metal cation, while the fragmented bonds display the weaker BH_4_^–^ interactions with the surrounding NH_3_ groups (dihydrogen bonds). H on NH_3_ is omitted for clarity. Color scheme: Y (gray), N (blue), B (green), H (pink), BH_4_^–^ (green tetrahedra).

The elastic and quasielastic contributions were extracted from the data by fitting the collected QENS spectra to eq 1, and using these contributions, the experimental EISFs were determined. These EISFs are compared to several plausible reorientational models in Figure 3a–c. The different reorientational motions are shown in Figure 3d–f, and their mathematical descriptions can be found in the Supporting Information or in refs (43−46). For Y(BH_4_)_3_, the best agreement between the experimental data and EISF models is found for a 2-fold rotation around its C_2_ axis or 3-fold rotation around its C_3_ axis, which is in good agreement with a recent NMR study.^47^ For BH_4_^–^, the EISF values for C_2_ and C_3_ rotations are identical, and it is thus not possible to determine which of the two mechanisms occurs solely from the QENS data. However, crystallographic data show that the BH_4_^–^ anion acts as a bridging ligand and coordinates to two yttrium cations in a linear arrangement with two hydrogen facing each of the yttrium cations; see Figure 4a.^47,48^ Given this arrangement, it is possible for the BH_4_^–^ anion to perform a 2-fold rotation around the C_2_ axis along the Y–BH_4_–Y axis without breaking any bonds, and thus a 2-fold rotation is more probable than a 3-fold rotation.^47^

In a similar manner to Y(BH_4_)_3_, the EISF for Y(BH_4_)_3_·3ND_3_ was determined, and as shown in Figure 2b, the best agreement is found for a C_2_ or C_3_ rotation of the BH_4_^–^ anion. In Y(BH_4_)_3_·3ND_3_, the BH_4_^–^ anions act as terminal ligands, where they coordinate via the face of the tetrahedron to the Y-cation; see Figure 4 b).^48^ Thus, the most likely reorientation is a 3-fold reorientation around the C_3_ axis.

For Y(BH_4_)_3_·7ND_3_, the best agreement between the experimental EISF and the EISF model is for a 2-fold rotation around the C_2_ axis or 3-fold rotation around the C_3_ axis; see Figure 2c. In Y(BH_4_)_3_·7ND_3_, the BH_4_^–^ anion is entirely surrounded by ammonia molecules, and no clear preferred axis of rotation is evident based on the crystal structure; see Figure 4c. Based on the EISF collected at different temperatures, it is however clear that there is more than one reorientational frequency since only two-thirds of the BH_4_^–^ anions are dynamically active on the instrument time scale at 100 K, while all anions are dynamically active at 250 K; see Figure 2c. In the crystal structure for Y(BH_4_)_3_·7ND_3_, there are multiple local environments for the BH_4_^–^ anion with a varying extent of dihydrogen bonding between H^δ+^ on ND_3_ and H^δ−^ on the BH_4_^–^, which results in a difference in the reorientational mobility.

### III.C Reorientational Energy Barriers for BH_4_^–^

**Figure 5:**
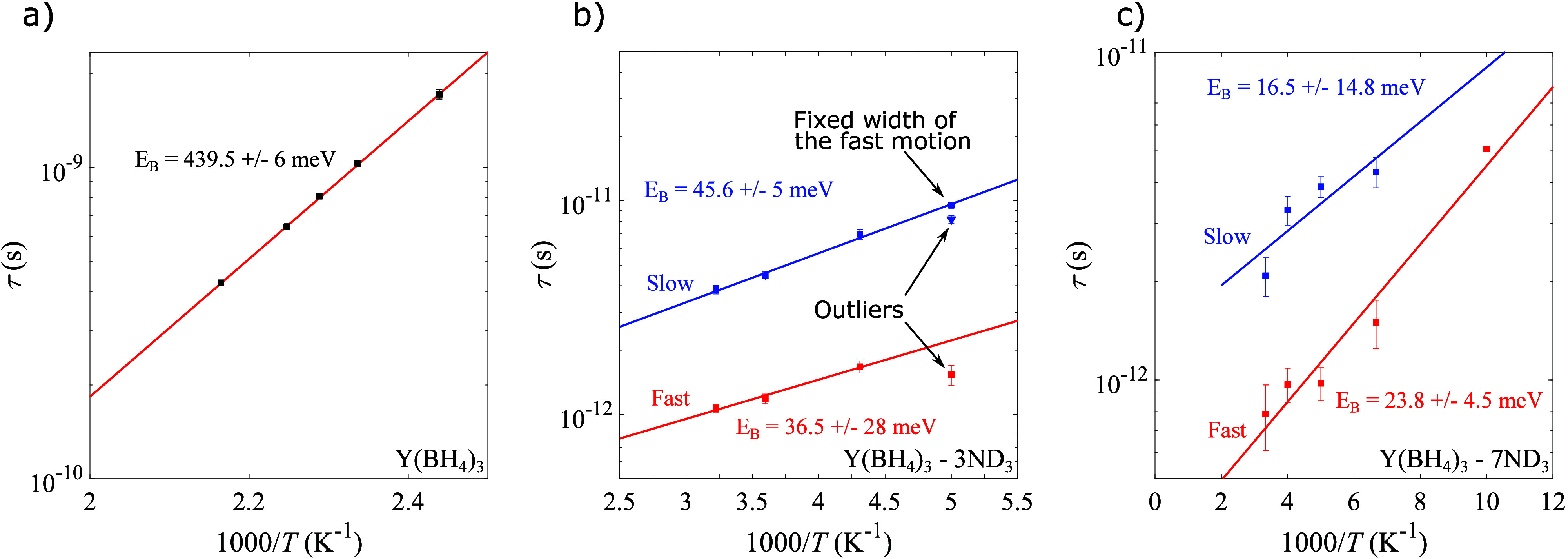
Temperature dependence of the relaxation time (τ) of the BH_4_^–^ anion in (a) Y(BH_4_)_3_, (b) Y(BH_4_)_3_·3ND_3_, and (c) Y(BH_4_)_3_·7ND_3_. The red and blue lines correspond to fits of the relaxation time to eq 3. In (b), the points marked as outliers were not used in the fits. The point marked as “Fixed width of the fast motion” was extracted from fits of the QENS spectrum at 200 K where the width of the fast motion was fixed to the estimated value from eq 3 using the widths at 232, 278, and 310 K. More information about this is given in the main text. Error bars in (a), (b), and (c) correspond to two standard deviations.

Using the Lorentzian widths (Γ) extracted from the fits of the QENS spectra to eq 1, the reorientational energy barriers (*E*_B_) were determined by fitting the relaxation time τ = 2ℏ/Γ to
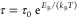
3over a wide temperature range as shown in Figure 5. Here *k*_B_ is the Boltzmann constant. The energy barrier for BH_4_^–^ anion reorientations in Y(BH_4_)_3_ is about 440 meV; see Figure 5a. As described in section III.A, fitting of the QENS spectra for Y(BH_4_)_3_·3ND_3_ and Y(BH_4_)_3_·7ND_3_ requires two Lorentzians (QENS components), suggesting a distribution in the reorientational mobility of the BH_4_^–^ anions in these two compounds, which henceforth will be referred to as slow and fast. The Lorentzian widths for the slow and the fast motion for Y(BH_4_)_3_·3ND_3_ extracted from the 200 K QENS spectrum were identified as outliers; see Figure 5 b). Using eq 3, the width of the fast motion at 200 K was estimated from the widths extracted from the QENS spectra at 232 K, 278 K, and 310 K. The estimated fixed width of the fast motion was then used to refit the 200 K QENS spectrum to extract the width of the slow motion at 200 K, which in turn was used to determine the energy barrier of the slow motion. For Y(BH_4_)_3_·3ND_3_, the energy barriers are about 35 meV for the faster motion and 45 meV for the slower motion, while for Y(BH_4_)_3_·7ND_3_, the energy barriers are about 15 and 25 meV for the faster and slower motions, respectively. This shows that the introduction of the neutral ligand can modify the local environment in such a way that the energy barrier of rotation changes by an order of magnitude. Using the determined values from the fits to eq 3, it is possible to estimate the relaxation time of the reorientational motion for each of the compounds at 300 K. For Y(BH_4_)_3_, this is estimated to be 2 × 10^–7^ s, while for the fastest reorientational motion of Y(BH_4_)_3_·3ND_3_ and Y(BH_4_)_3_·7ND_3_, it is estimated to be 1 × 10^–12^ s and 7 × 10^–13^, respectively. This shows that the addition of neutral ammonia ligands to Y(BH_4_)_3_ can significantly change the dynamics of the BH_4_^–^ anion. The increase in the reorientational mobility with increasing NH_3_ concentration is likely due to the decreased interaction with the Y^3+^ cation stemming from the increased screening of the cation by the NH_3_ ligands. For *x* = 0 the BH_4_^–^ anion coordinates to two Y^3+^ cations, while the anion coordinates to one cation for *x* = 3 and to zero cations in *x* = 7; see Figure 4.^36,49^

### III.D Reorientational Dynamics of the NH_3_ Ligand

**Figure 6:**
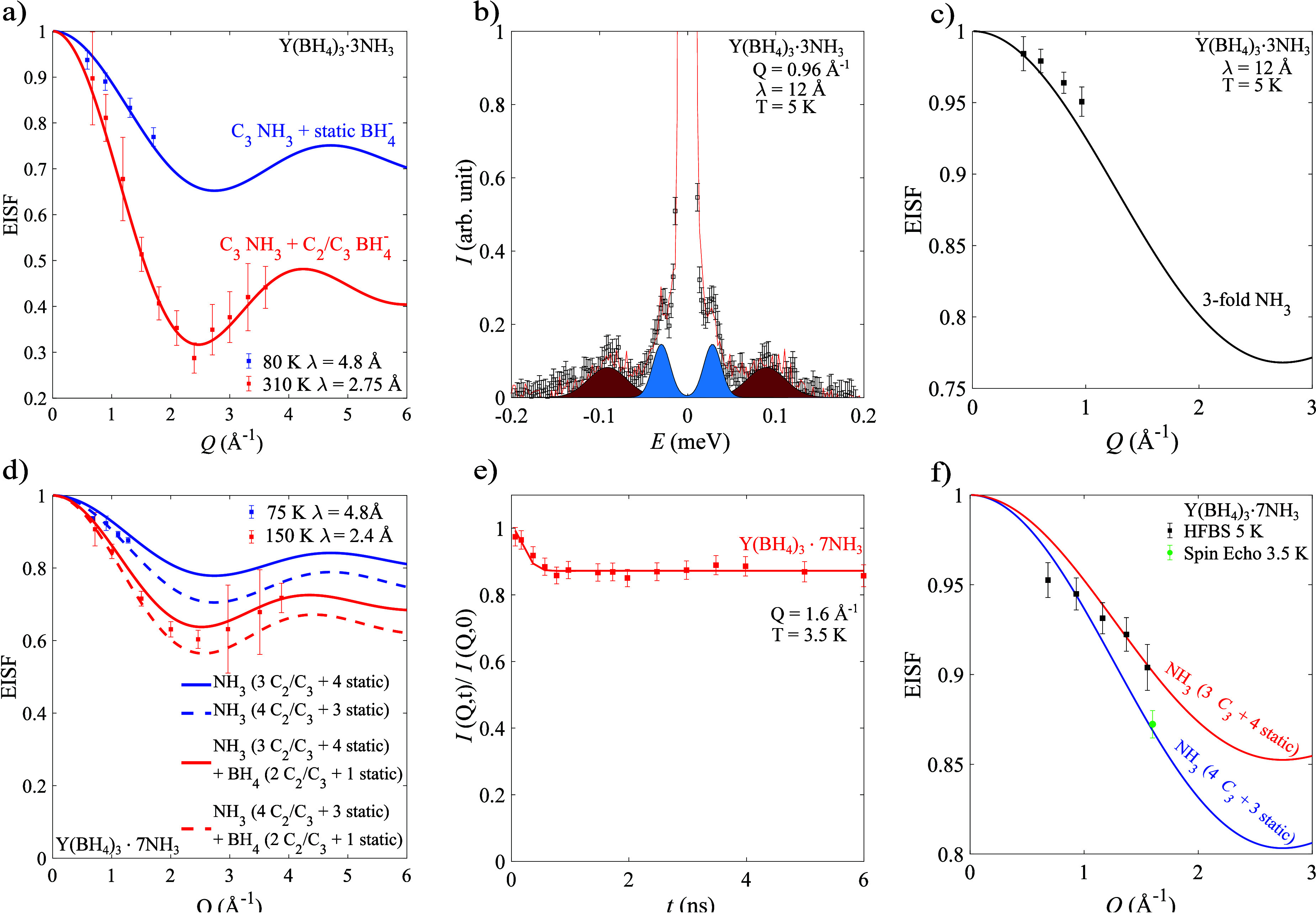
(a, d) EISFs for (a) Y(BH_4_)_3_·3NH_3_ and (d) Y(BH_4_)_3_·7NH_3_ compared to EISF models for each respective system. (b) Low-temperature (5 K) QENS spectra and fit for Y(BH_4_)_3_·3NH_3_. The components corresponding to the quantum mechanical tunneling peaks are highlighted. (c, f) Tunneling EISF for (c) Y(BH_4_)_3_·3NH_3_ and (f) Y(BH_4_)_3_·7NH_3_. (e) Low-temperature (3.5 K) NSE data with corresponding fit for Y(BH_4_)_3_·7NH_3_. Error bars in (a), (c), (d), (e), and (f) correspond to two standard deviations, while error bars in (b) correspond to one standard deviation.

To explore the dynamics of the NH_3_ ligand, QENS spectra for Y(BH_4_)_3_·3NH_3_ and Y(BH_4_)_3_·7NH_3_ were collected at a few selected temperatures (80 K and 310 K for Y(BH_4_)_3_·3NH_3_, and 75 and 150 K for Y(BH_4_)_3_·7NH_3_). At 80 K, the experimental EISF for Y(BH_4_)_3_·3NH_3_ is expected to only contain NH_3_ dynamics, since the dynamics of the BH_4_^–^ anion are too slow to be resolved at this temperature. The experimental EISF agrees well with an EISF model where the NH_3_ ligand performs 3-fold rotations around its C_3_ axis, while the BH_4_^–^ anions are frozen (static on the experimental time scale); see Figure 6a. It should be noted that while 2-fold and 3-fold rotations around the C_2_ and C_3_ axes in tetrahedral molecules or ions have identical EISF models, the same is not true for the case of NH_3_, and it is therefore possible to exclude a 2-fold rotation. A 3-fold rotation also agrees well with crystallographic studies of Y(BD_4_)_3_·3ND_3_, which suggest that the nitrogen atom of the NH_3_ ligand faces yttrium while the 3 hydrogens are facing away, allowing the molecule to perform 3-fold rotations without breaking any bonds.^48^ The experimental EISF was also extracted for Y(BH_4_)_3_·3NH_3_ at 310 K where both the NH_3_ ligands and the BH_4_^–^ anions are expected to be dynamically active. As shown in Figure 6a, a good agreement is found between the experimental EISF at 310 K and the EISF model that takes into account both 3-fold rotation of the NH_3_ ligands and a 3-fold rotation around the C_3_ axis for the BH_4_^–^ anions as determined from the QENS experiments on Y(BH_4_)_3_·3ND_3_; see Figure 3b.

For Y(BH_4_)_3_·7NH_3_, the crystal structure implies that, as for Y(BH_4_)_3_·3NH_3_, the NH_3_ ligand is likely to perform reorientations around its C_3_ axis since the nitrogen atom is facing the yttrium while the 3 hydrogens are facing away. The experimental EISF extracted from QENS spectra collected at 75 and 150 K are presented with the closest EISF models in Figure 6d. For 75 K, it is expected that only the NH_3_ ligands are active, while at 150 K it is expected that both the NH_3_ ligands and the BH_4_^–^ anions are dynamically active on the instrument time scale. For the EISF extracted from the 75 K QENS spectrum, the best agreement is found for a model where the NH_3_ ligands perform 3-fold rotations around the C_3_ axis. However, the model suggests that only 3 or 4 of the 7NH_3_ ligands are dynamically active at 75 K; see Figure 6d. While the data agree slightly better with the model suggesting 4 dynamically active NH_3_ ligands, the differences are not significant enough to exclude the model for 3 dynamically active NH_3_ ligands. At 150 K, the experimental EISF suggests that, in addition to the active NH_3_ ligands, the BH_4_^–^ anions are also dynamically active. As suggested by the QENS data for Y(BH_4_)_3_·7ND_3_, it is likely that only 2 of the 3 BH_4_^–^ anions are active on the instrument time scale at 150 K; see Figure 3c. Furthermore, the QENS data from Y(BH_4_)_3_·7ND_3_ suggests that the BH_4_^–^ anions perform 2-fold/3-fold rotations around the C_2_/C_3_ axis. A good agreement is found between the experimental EISF for Y(BH_4_)_3_·7NH_3_ with a model that takes into account 4 dynamically active NH_3_ ligands performing 3-fold rotations around their C_3_ axes together with 2 dynamically active BH_4_^–^ anions performing 2-fold/3-fold rotations around the C_2_/C_3_ axis. However, similar to the QENS data at 75 K, the data also agree well with a model that takes into account 3 dynamically active NH_3_ ligands rather than 4. Thus, it can be concluded that only some of the NH_3_ ligands are dynamically active at these temperatures, but the exact number is uncertain. From the crystal structure of Y(BH_4_)_3_·7NH_3_, it is clear that there are multiple environments for the NH_3_ ligands, which is likely to yield a distribution of reorientational mobilities. To fully determine the reorientational dynamics of the NH_3_ ligands in Y(BH_4_)_3_·7NH_3_, a dedicated QENS study using Y(BD_4_)_3_·7NH_3_ to isolate the NH_3_ dynamics is needed, but this is considered to be beyond the scope of this article.

### III.E Quantum Mechanical Rotational Tunneling of the NH_3_ Ligand

In addition to the reorientational dynamics described above, QENS measurements at 5 K revealed that both Y(BH_4_)_3_·3NH_3_ and Y(BH_4_)_3_·7NH_3_ exhibit quantum mechanical tunneling peaks, as shown in Figure 6b and in Figure S3a in the Supporting Information. While both compounds exhibit two sets of tunneling peaks, the tunneling energies, *E*_T_, for Y(BH_4_)_3_·3NH_3_ (≈30 μeV and ≈90 μeV) are much larger than for Y(BH_4_)_3_·7NH_3_ (≈ 1 μeV and ≈2.5 μeV). QENS and NSE measurements of selectively deuterated Y(BH_4_)_3_·3ND_3_ and Y(BH_4_)_3_·7ND_3_ samples revealed that the tunneling is related to NH_3_, since no tunneling was detected after exchanging NH_3_ for ND_3_; see Figure S3b in the Supporting Information. By fitting the elastic peak with one δ-function and one Gaussian function per tunneling peak (all convoluted with the resolution function), the tunneling EISF could be determined from the QENS data for Y(BH_4_)_3_·3NH_3_ and Y(BH_4_)_3_·7NH_3_. For an NH_3_ ligand undergoing 3-fold rotational tunneling around its C_3_ axis, the tunneling EISF is
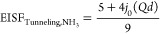
4where *j*_0_ is the spherical Bessel function of zeroth order and *d* is the H jump distance.^50^ The tunneling EISF functions for Y(BH_4_)_3_·3NH_3_ and Y(BH_4_)_3_·7NH_3_ also must take into account the static hydrogen from the BH_4_^–^ anions, which do not undergo rotational tunneling, resulting in a combined tunneling EISF function EISF_tot_ = (1 – *z*)EISF_NH_3__ + *z*, where *z* is the fraction of static hydrogen atoms which do not exhibit tunneling on the instrument time scale. In Figure 6c,f, the experimental tunneling EISF for Y(BH_4_)_3_·3NH_3_ and Y(BH_4_)_3_·7NH_3_ are shown together with the closest models. For Y(BH_4_)_3_·3NH_3_, the best agreement with the experimental data is found for a model where all of the NH_3_ ligands undergo 3-fold rotational tunneling. Similar to what was found from the QENS spectra for Y(BH_4_)_3_·7NH_3_ at 75 K and 150 K, the best agreement with the experimental data is found for a model where 3 or 4 out of 7NH_3_ ligands undergo 3-fold rotational tunneling in Y(BH_4_)_3_·7NH_3_. However, the uncertainty of the tunneling EISF for Y(BH_4_)_3_·7NH_3_ is high because the lowest lying tunneling peak pair has a large overlap with the main elastic peak and is thus difficult to accurately quantify. The 4 or 3 remaining “static” NH_3_ ligands in Y(BH_4_)_3_·7NH_3_ either undergo tunneling at lower energies and are thus indistinguishable from the main elastic peak or do not perform rotational tunneling at all.

To investigate if there are tunneling peaks at lower energies, NSE measurements were made to probe slower time scales (smaller energies) for Y(BD_4_)_3_·7NH_3_ (at *Q* = 1.15 Å^–1^), Y(BH_4_)_3_·7ND_3_ (at *Q* = 1.15 Å^–1^), and Y(BH_4_)_3_·7NH_3_ (at *Q* = 1.6 Å^–1^). As shown in Figure 6e and in Figure S3b in the Supporting Information, both Y(BD_4_)_3_·7NH_3_ and Y(BH_4_)_3_·7NH_3_ exhibit a rapid decay in the intensity between ≈0.1 ns and ≈1 ns, which corresponds to active dynamics. At times larger than ≈1 ns, the intensity remains constant, and the value of this intensity plateau corresponds to the tunneling EISF at the *Q*-value for which the spin echo measurement was made. For Y(BH_4_)_3_·7ND_3_, no change in the NSE intensity is observable, suggesting that no relaxation process occurs on the time scale of the NSE-spectrometer for Y(BH_4_)_3_·7ND_3_; see Figure S3b in the Supporting Information. This confirms that the observed tunneling is related to the NH_3_ ligands and not to the BH_4_^–^ anions. The NSE data are well described by a model with two sets of Gaussian-broadened tunneling peaks with fwhm (full width at half maximum) line widths (*w*) that are centered at ±*E*_T_ using the values of *w* and *E*_T_ determined from the QENS spectra at 5 K; see Figure S3a in the Supporting Information. The fits of the NSE data for Y(BH_4_)_3_·7NH_3_ and Y(BD_4_)_3_·7NH_3_ are shown as solid lines in Figure 6e and Figure S3b in the Supporting Information, respectively. Since no additional tunneling peak pairs besides the two detected by the QENS experiment at 5 K are required to describe the NSE data, it can be concluded that the 4 or 3 remaining NH_3_ ligands do not exhibit tunneling even on the significantly slower times scales probed by NSE. From the fits of the NSE data, the tunneling EISF (height of the plateau) was determined for Y(BD_4_)_3_·7NH_3_ (at *Q* = 1.15 Å^–1^) and Y(BH_4_)_3_·7NH_3_ (at *Q* = 1.6 Å^–1^). These experimental tunneling EISF values agree well with only the tunneling EISF model with 4 tunneling and 3 static NH_3_ ligands, strongly suggesting that 4 out of 7NH_3_ ligands undergo tunneling; see Figure 6f). As NSE probes time scales slower than QENS, the EISF determined from NSE for Y(BD_4_)_3_·7NH_3_ and Y(BH_4_)_3_·7NH_3_ should be given more weight than the EISF from the QENS data where the innermost tunneling peak pair overlaps with the main elastic peak. Because the tunneling energy barriers are determined by the local surroundings of the NH_3_ ligand, this implies a significant difference in their local environment, which is in good agreement with the crystal structure for Y(BH_4_)_3_·7NH_3_.^48^

## IV Conclusions

Using QENS, we have shown that the BH_4_^–^ rotational dynamics in Y(BH_4_)_3_·*x*NH_3_ (*x* = 0, 3, and 7) is significantly influenced by changing the number of NH_3_ ligands. At 300 K, the average time between jumps for the BH_4_^–^ anion is 2 × 10^–7^ s for Y(BH_4_)_3_ while for Y(BH_4_)_3_·3ND_3_ and Y(BH_4_)_3_·7ND_3_, it is 1 × 10^–12^ s and 7 × 10^–13^ s, respectively. Comparisons between the experimental EISF and EISF models suggest that the BH_4_^–^ anion in Y(BH_4_)_3_ performs 2-fold reorientations around the C_2_ axis, while in Y(BH_4_)_3_·3ND_3_, it performs 3-fold reorientations around the C_3_ axis. For Y(BH_4_)_3_·7ND_3_, the experimental EISF suggests that the BH_4_^–^ anion performs either a 2-fold reorientation around the C_2_ axis or a 3-fold reorientation around the C_3_ axis. Quasielastic neutron scattering measurements also suggest that the NH_3_ ligands perform 3-fold reorientations around the C_3_ axis in Y(BH_4_)_3_·3NH_3_ and Y(BH_4_)_3_·7NH_3_. In addition to the classical reorientation dynamics, it was found that the NH_3_ ligands in Y(BH_4_)_3_·3NH_3_ and Y(BH_4_)_3_·7NH_3_ exhibit 3-fold quantum mechanical rotational tunneling at 5 K. Furthermore, QENS and NSE experiments also revealed that there is a distribution of reorientional mobilities of the BH_4_^–^ anions and NH_3_ ligands in both Y(BH_4_)_3_·3NH_3_ and Y(BH_4_)_3_·7NH_3_. This research demonstrates how introducing a neutral ligand can greatly alter the dynamics in the compounds, which may prove important for rational design of future solid-state room-temperature superionic conductors based on Li, Na, and Mg borohydrides with ligands such as NH_3_ or CH_3_NH_2_, where flexible structures and dynamics play an important role in enhancing the cation conductivity.

## Supporting Information Available

Information about the crystal structures, the *Q*-dependence of the fwhm, model EISF functions, and additional low-temperature QENS and NSE data (PDF)
