# Supplementary material for: Reorientational Dynamics in Y(BH4)3·xNH3 (x = 0, 3, and 7): The Impact of NH3 on BH4– Dynamics
Source: J Phys Chem C Nanomater Interfaces. 2024 Mar 9;128(11):4431–9. doi: 10.1021/acs.jpcc.4c00265 (PMC10961835; doi:10.1021/acs.jpcc.4c00265)
Supplement: Supplementary file 1 — jp4c00265_si_001.pdf [file jp4c00265_si_001.pdf]

Supporting Information for:

Reorientational Dynamics in $Y(BH_4)_3 \cdot x NH_3$ (x = 0, 3, and 7) – The Impact of NH_3 on the BH_4^- Dynamics

J. B. Grinderslev,[1] U. Häussermann,[2] T. R. Jensen,[1] A. Faraone,[3] M. Nagao,[3,4,5] M. Karlsson,[6] T. J. Udovic,[3,4] and M. S. Andersson[7,*]

[1]*Interdisciplinary Nanoscience Center (iNANO) and Department of Chemistry, Aarhus University, DK-8000, Denmark*

[2]*Department of Materials and Environmental Chemistry, Stockholm University, SE-10691 Stockholm, Sweden*

[3]*NIST Center for Neutron Research, National Institute of Standards and Technology, Gaithersburg, Maryland 20899-6102, United States*

[4]*Department of Materials Science and Engineering, University of Maryland, College Park, MD 20742-2115, United States*

[5]*Department of Physics and Astronomy, University of Delaware, Newark, DE 19716, United States*

[6]*Department of Chemistry and Chemical Engineering, Chalmers University of Technology, Göteborg SE-412 96, Sweden*

[7]*Department of Chemistry - Ångström Laboratory, Uppsala University, Box 538, SE-751 21 Uppsala, Sweden*

*E-mail: mikael.andersson@kemi.uu.se (M. S. Andersson).

1 Crystal structure of $Y(BH_4)_3 \cdot x NH_3$ (x = 0, 3, and 7)

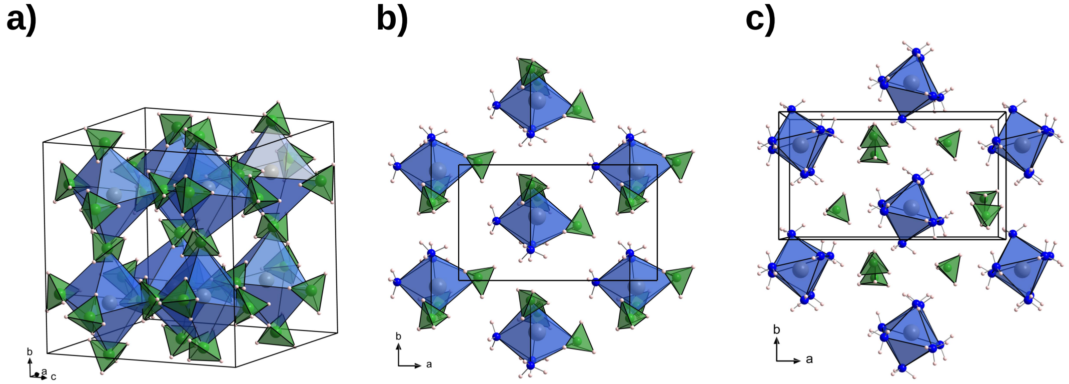

Fig. S1 Crystal structures of a) α-$Y(BH_4)_3$, b) $Y(BH_4)_3 \cdot 3NH_3$ and c) $Y(BH_4)_3 \cdot 7NH_3$. α-$Y(BH_4)_3$ is a three-dimensional structure with connected octahedral units of $[Y(BH_4)_6]$ through bridging BH_4^- groups. $Y(BH_4)_3 \cdot 3NH_3$ is a molecular structure with neutral units of $[Y(NH_3)_3(BH_4)_3]$. $Y(BH_4)_3 \cdot 7NH_3$ consists of the ionic complexes $[Y(NH_3)_7]^{3+}$, which are charge-balanced by BH_4^-. Color scheme: Y (grey), N (blue), B (green), H (Pink), BH_4^- (green tetrahedra), local Y coordination (blue polyhedra).

2 FWHM as a function of Q for $Y(BH_4)_3 \cdot x ND_3$ (x = 0, 3, and 7)

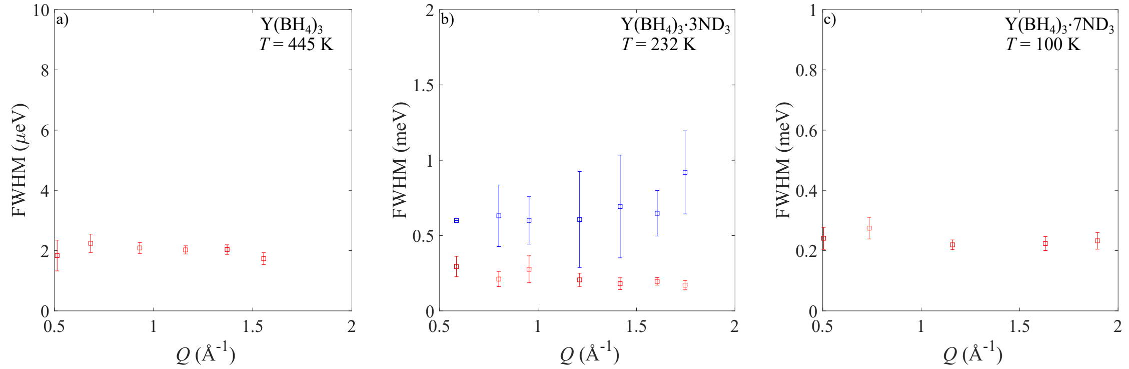

Fig. S2 FWHM of the Lorentzian widths extracted from fitting for a) $Y(BH_4)_3$ at 100 K, b) $Y(BH_4)_3 \cdot 3ND_3$ at 232 K, and c) $Y(BH_4)_3 \cdot 7ND_3$ at 100 K. Error bars are two standard deviations.

3 Mathematical expressions of the EISF model curves for NH_3 and BH_4^-

The EISF models C_2 or C_3 reorientation,[S1] tetrahedral tumbling and [S2] cubic tumbling[S3] for a tetrahedral molecule or ion such as BH_4^- are given by:

$$\mathrm{EISF}_{C_2/C_3,\ \mathrm{BH}_4^-} = \frac{1 + j_0(Qd)}{2}, \tag{1}$$

$$\mathrm{EISF}_{\mathrm{tetrahedral},\ \mathrm{BH}_4^-} = \frac{1 + 3j_0(Qd)}{4}, \tag{2}$$

$$\mathrm{EISF}_{\mathrm{cubic},\ \mathrm{BH}_4^-} = \frac{1 + 3j_0(Qd/\sqrt{2}) + 3j_0(Qd) + j_0(Qd\sqrt{3/2})}{8}, \tag{3}$$

where d is the jump distance and $j_0 = \sin(x)/x$ is the zeroth-order spherical Bessel function. For a trigonal pyramidal molecule such as NH_3 the EISF model for C_3 reorientation[S4] is given by:

$$\mathrm{EISF}_{C_3,\ \mathrm{NH}_3} = \frac{1 + 2j_0(Qd)}{3}, \tag{4}$$

For the specific case of NH_3 and BH_4^-, d is the same as the hydrogen-hydrogen distance in the respective molecule/ion (1.64 Å in NH_4^+, 1.96 Å in BH_4^-).

4 Quantum mechanical tunneling

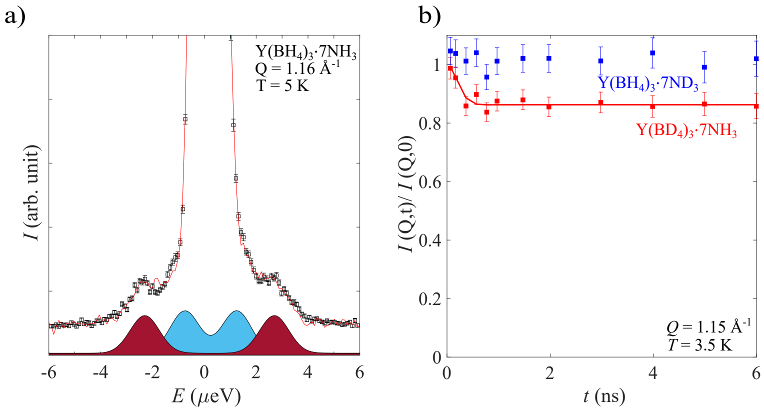

Fig. S3 a) Low-temperature (5 K) QENS spectra and fit for $Y(BH_4)_3{\cdot}7NH_3$. The components corresponding to the quantum mechanical tunneling peaks are highlighted. b) Low temperature (3.5 K) NSE data with corresponding fits for $Y(BH_4)_3{\cdot}7ND_3$ as well as $Y(BD_4)_3{\cdot}7NH_3$. Error bars in a) correspond to one standard deviation, while error bars in b) correspond to two standard deviations.

References

S1. Yildirim, T.; Gehring, P. M.; Neumann, D. A.; Eaton, P. E.; Emrick, T. Neutron-scattering investigation of molecular reorientations in solid cubane. *Phys. Rev. B* **1999**, *60*, 314–321

S2. Sköld, K. Effects of molecular reorientation in solid methane on the quasielastic scattering of thermal neutrons. *J. Chem. Phys.* **1968**, *49*, 2443–2445

S3. Rush, J. J.; de Graaf, L. A.; Livingston, R. C. Neutron scattering investigation of the rotational dynamics and phase transitions in sodium and cesium hydrosulfides. *J. Chem. Phys.* **1973**, *58*, 3439–3448.

S4. Bée, M. *Quasielastic Neutron Scattering*; Adam Hilger, Bristol, 1988
